# Tramadol and O-demethyltramadol disposition in humans: a pooled study

**DOI:** 10.1186/cc9767

**Published:** 2011-03-11

**Authors:** K Allegaert, U Stamer, B Anderson, S Holford, A Rochette, I Troconiz, R Pedersen, N Holford

**Affiliations:** 1 University Hospitals Leuven, Belgium; 2 University of Bonn, Germany; 3 University of Auckland, New Zealand; 4 Hopital Lapeyronie, Montpellier, France; 5 University of Navarra, Pamplona, Spain; 6 University of Southern Denmark, Odense, Denmark

## Introduction

To study the use of size, maturation and CYP2D6 genotype score as predictors of i.v. tramadol (M) disposition throughout human life, published observations were pooled [1-6].

## Methods

M and O-demethyltramadol (M1) observations in 295 subjects (25 weeks postmenstrual age to 84.8 years) were available for population PK analysis (NON-MEM, two-compartment model for M and two additional compartments for M1). Covariates were weight, age, sex, disease (healthy/patient) and CYP2D6 genotype score. A sigmoid maturation model was used to describe changes in M (CLPO + CLPM), M1 formation (CLPM) and M1 elimination (CLMO) clearance. Phenotype-based and genotype-based models were used to distinguish poor CLPM subjects.

## Results

Differences in M disposition between children and adults were largely accounted for by maturation and size. CKLPM (TM50 40.3 weeks, Hill 9.09) and CLPO (TM50 39.1 weeks, Hill 5.8) display fast maturation, while CLMO matures slower. The phenotype-based mixture model estimated that 8.6 were slow metabolizers (18.3% of normal CLPM). Genotype-based estimates were also lower (68%) but not all subjects with a low CYP2D6 score were in the poor metabolizer group.

## Conclusions

Maturation of M elimination occurs early with 50% of adult values at full-term age. Maturation and age are key predictors, while CYP2D6 genotype score only explains some of the variability in M disposition.
